# Supplementary material for: Thumbs up: movements made by the thumb are smoother and larger than fingers in finger-thumb opposition tasks
Source: PeerJ. 2018 Oct 18;6:e5763. doi: 10.7717/peerj.5763 (PMC6196073; doi:10.7717/peerj.5763)

Algorithm for determination of onset and end of movements

Onset and end of movement determined

We used velocity profile to calculate the onset and end of movement for thumb and fingers separately with the following steps

Step1: We found the maximum value in the velocity profile.
Step2: We travelled backward in time from the maximum value of velocity, until the value becomes less than 5 % of the maximum velocity. The successive value is taken as the onset of movement.

for example, if maximum value in the velocity profile is found at 61 then travelling backward in time from 61, if 47th value is less than 5% of max value. Then the onset of movement was taken from 48th value.

Step3: We travelled forward in time from the maximum value of velocity, until the value becomes less than 5 % of the maximum velocity. The predecessor value is taken as the end of movement.

Similarly, if 88th value is less than 5% of max value (61). Then the end of movement is taken till 87th value.

Total movement range will be from 48 to 87th value.

Above algorithm is suitable for cases when there are no glitches on the displacement profile. But lets say for cases there are some glitches like one shown below

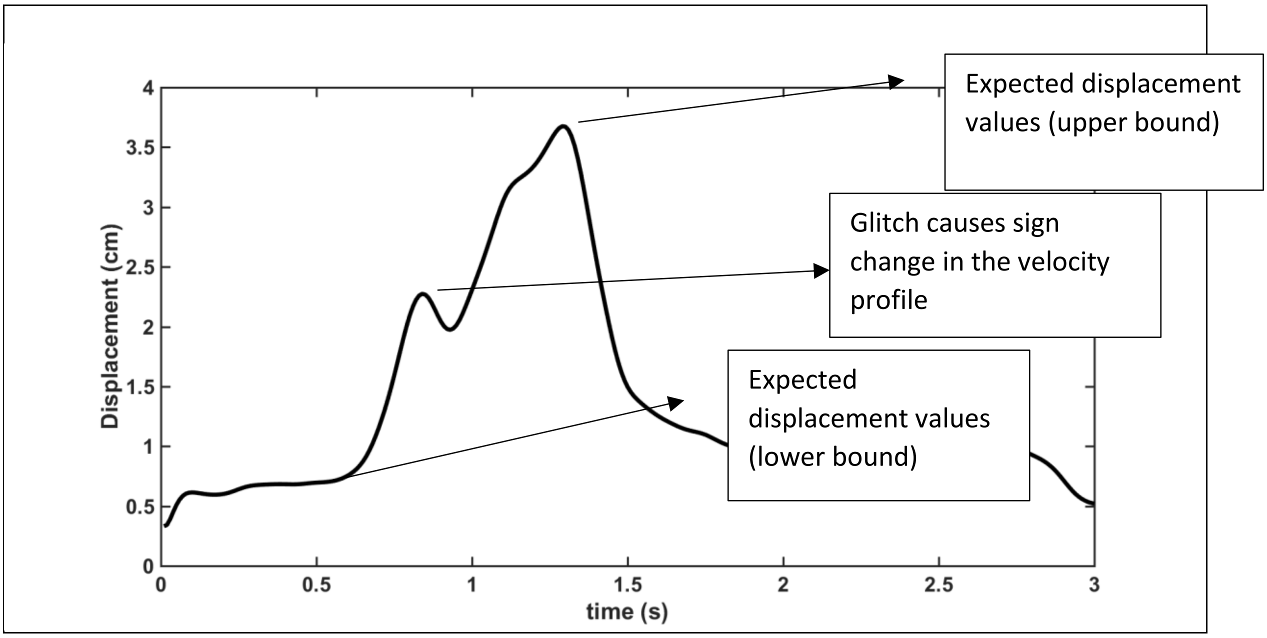

Corresponding velocity profile would be

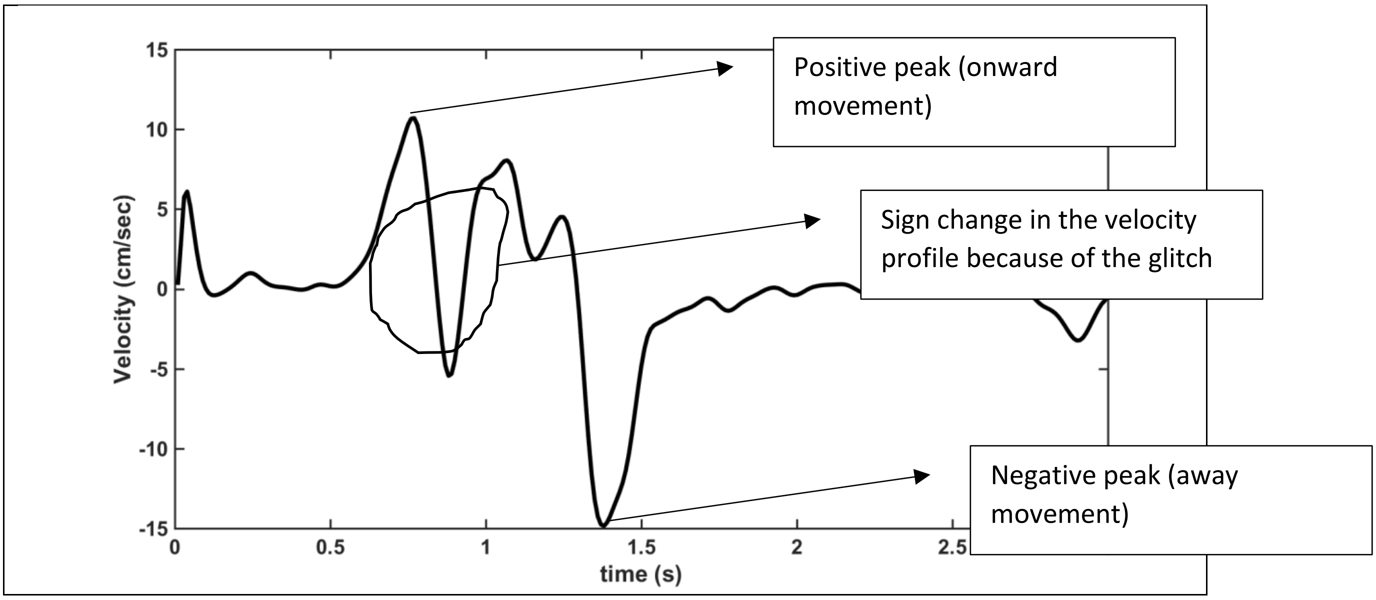

Expected value of displacement:

Visually inspecting the whole of the displacement and velocity profiles, we were able see that glitches occurred towards the end of the movement as the case described above. The expected values of displacement for lower and upper bound was determined as shown below.

For cases like these, there were false detection of onset and end of movement. Here in the above example glitch happens half the way in the displacement profile. This actually resulted in finding false end of movement at .85 secs rather than around 1.3 secs (as can be seen in displacement profile).

Lower bound value:

The velocity values between -0.5 to +0.5 cm /sec in the velocity profile before the max of the velocity was determined. The corresponding values in the displacement profile was averaged to obtain the lower bound value.

Iterative check after we calculate Onset of movement

1. t_{on} is the calculated time of onset of movement. The corresponding value in displacement profile is calculate disp(t_{on}). This value is compared with lower bound value

> Lw_disp_bound - disp(t_{on}) <0.1 then the calculated onset of movement is the actual value
>
> Lw_disp_bound - disp(t_{on}) >0.1. Then from the t_{on} the less than 5% value is once again checked till the condition is satisfied.

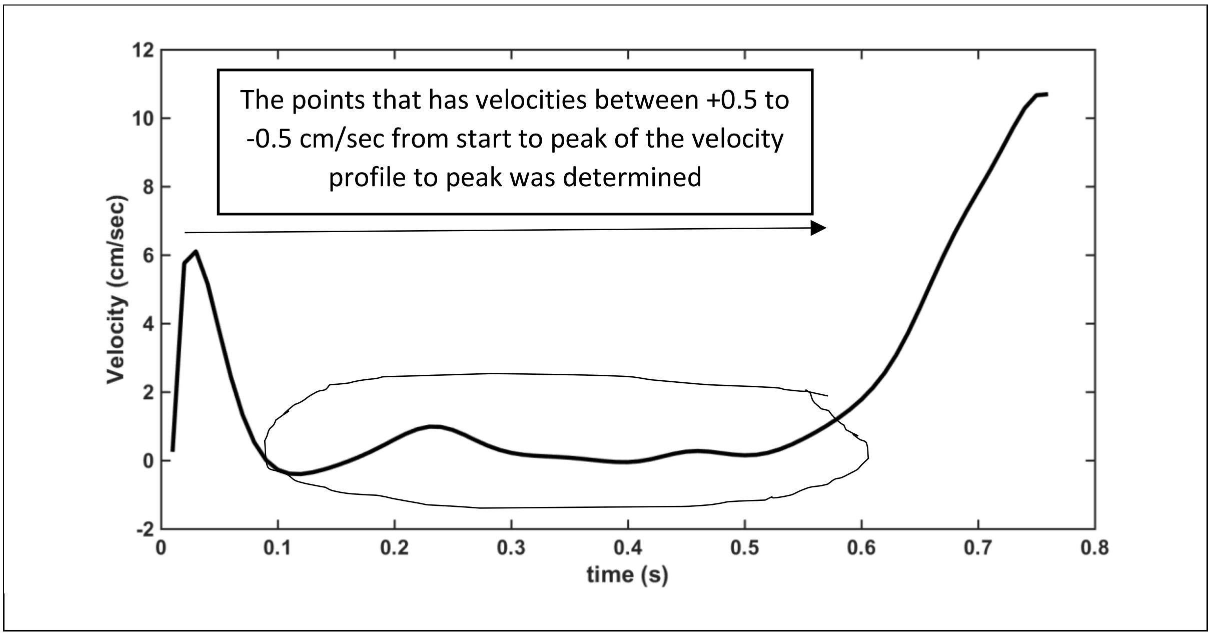

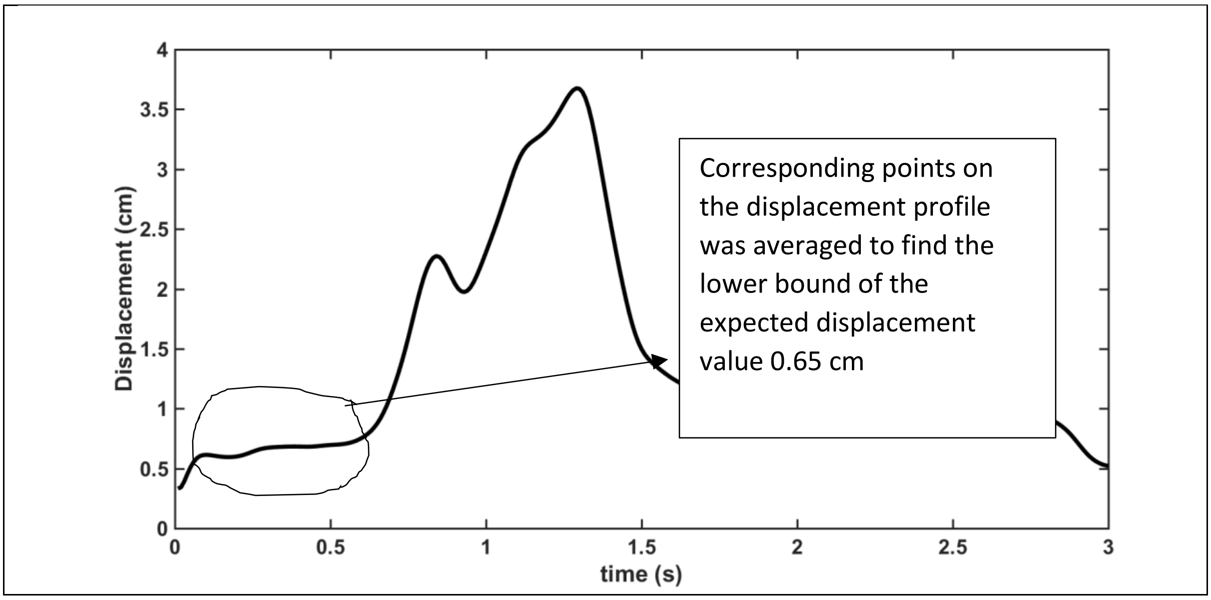

Upper bound value:

In the velocity profile, when travelling from negative peak towards the positive peak (backward in time), the first sign change was detected. This corresponding value in the displacement profile was considered as upper bound value

Iterative check after we calculate end of movement

1. t_{ed} is the calculated time of end of movement. The corresponding value in displacement profile is calculate disp(t_{ed}). This value is compared with Upper bound value

Up_disp_bound - disp(t_{ed}) <0.1 then the calculated end of movement is the actual value

Up_disp_bound - disp(t_{ed}) >0.1. Then from the t_{ed} the less than 5% value are once again checked till the condition is satisfied.

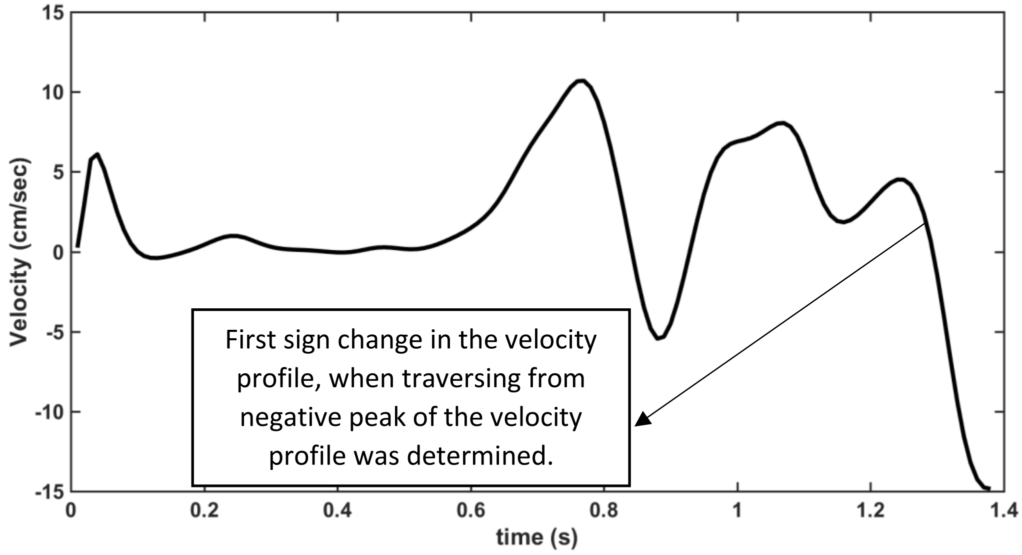

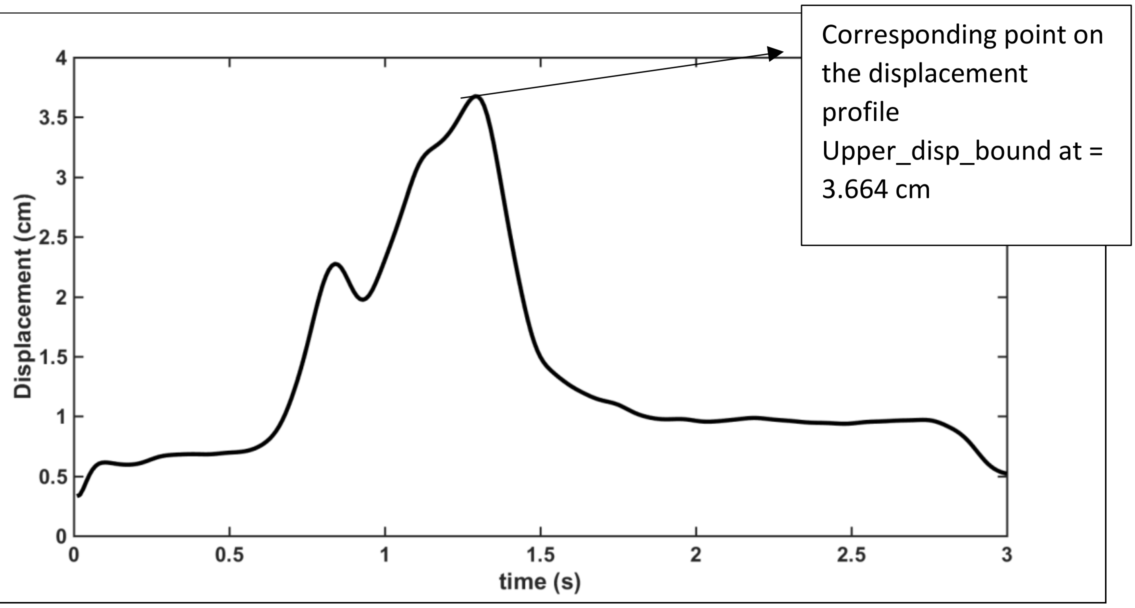

We used the minimum value in the velocity (negative maximum) to segregate the onward movement.

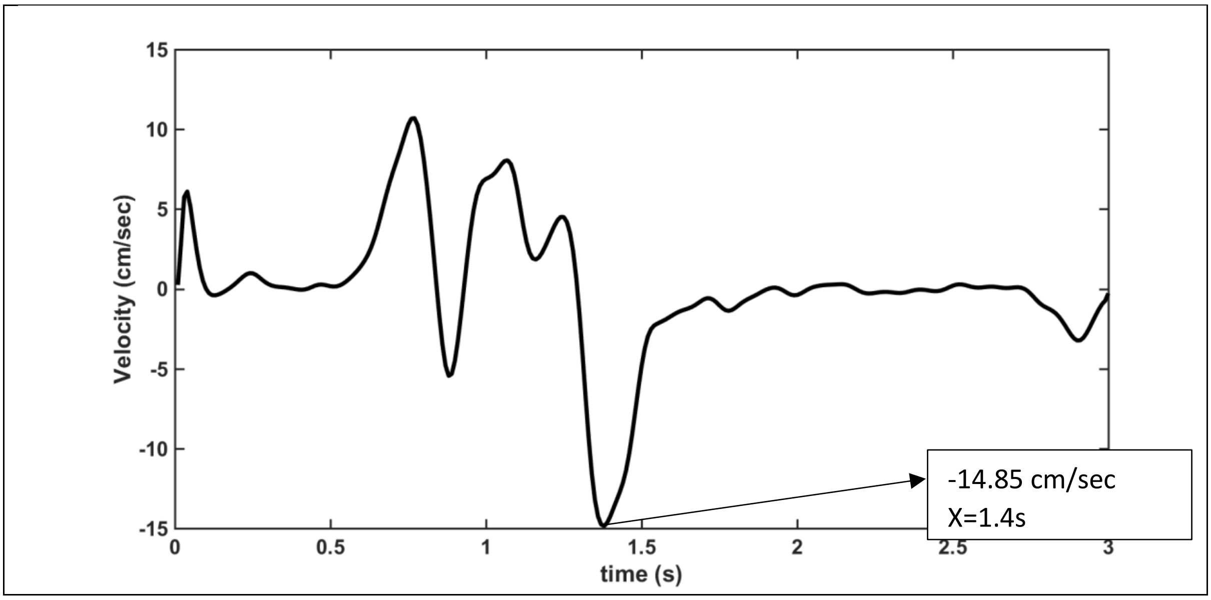

We found the maximum velocity from the segregated profile.

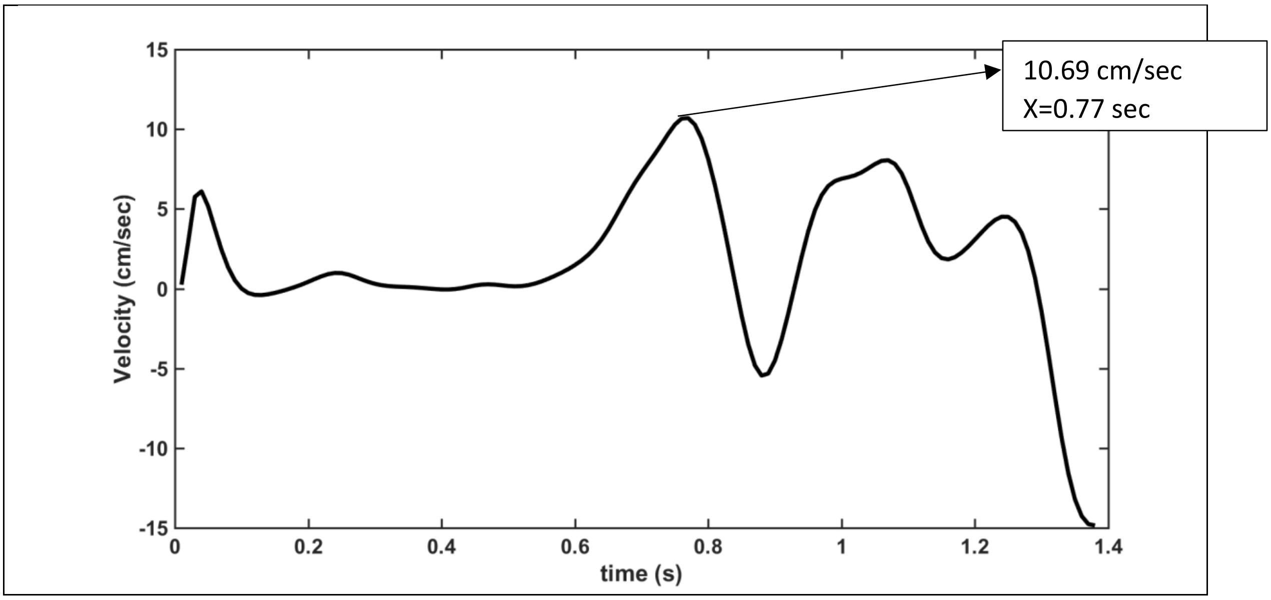

From the peak velocity we travelled backward in time from the max value (10.6 cm/sec) to find first time when the velocity became less than 5% of its max value, that's 0.5345

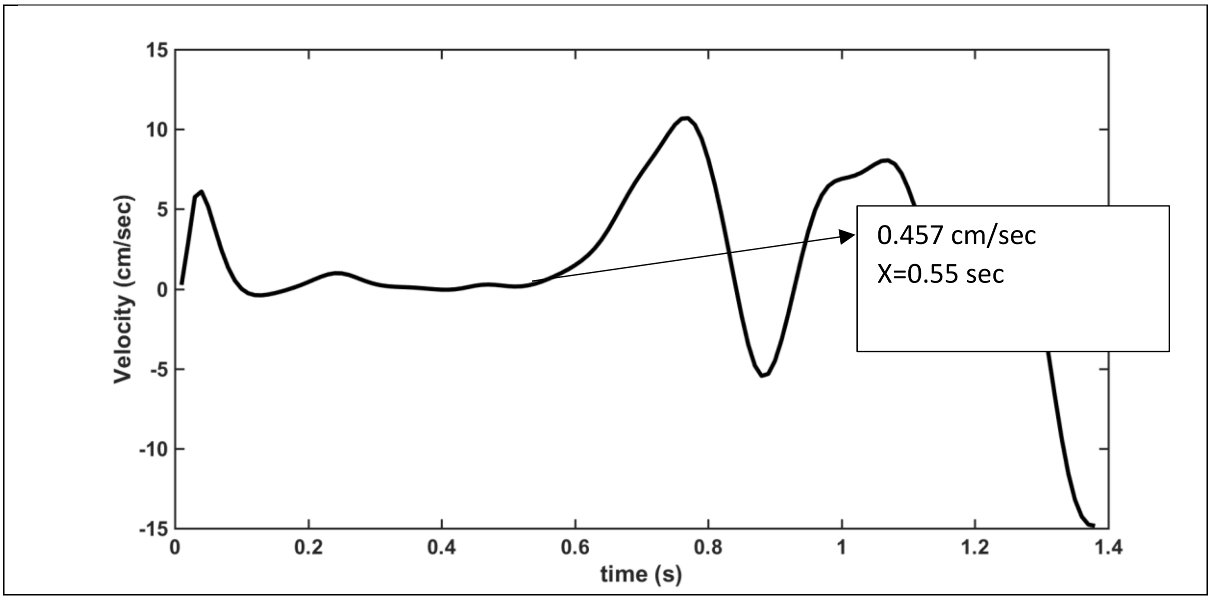

Onset movement was determined as (time of max velocity 0.77s)- first value of less than 5% of max velocity (22nd from top= 0.22 s) = 0.55s +0.01 =0.56

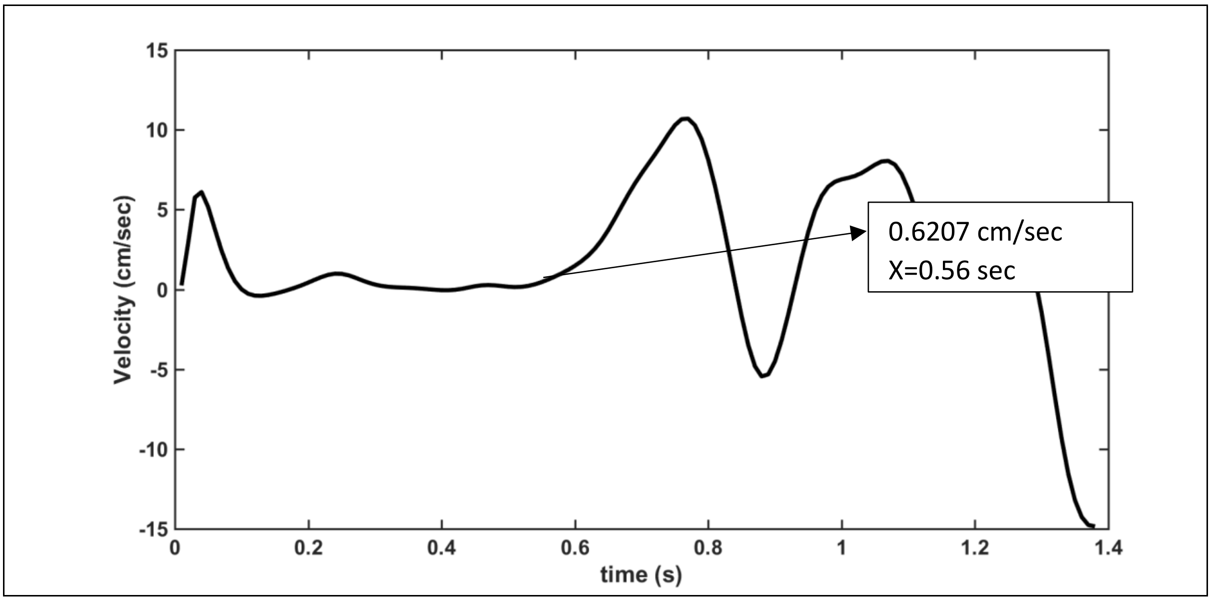

This will be the first-time velocity is greater than 5% of max velocity at 0.53 sec (onset of movement)

Here the condition is checked to

0.6537 ~ disp(0.56) = 0.657 ~ 0.712 = 0.029 < 0.1 therefore this point is the actual onset of movement

End of movement:

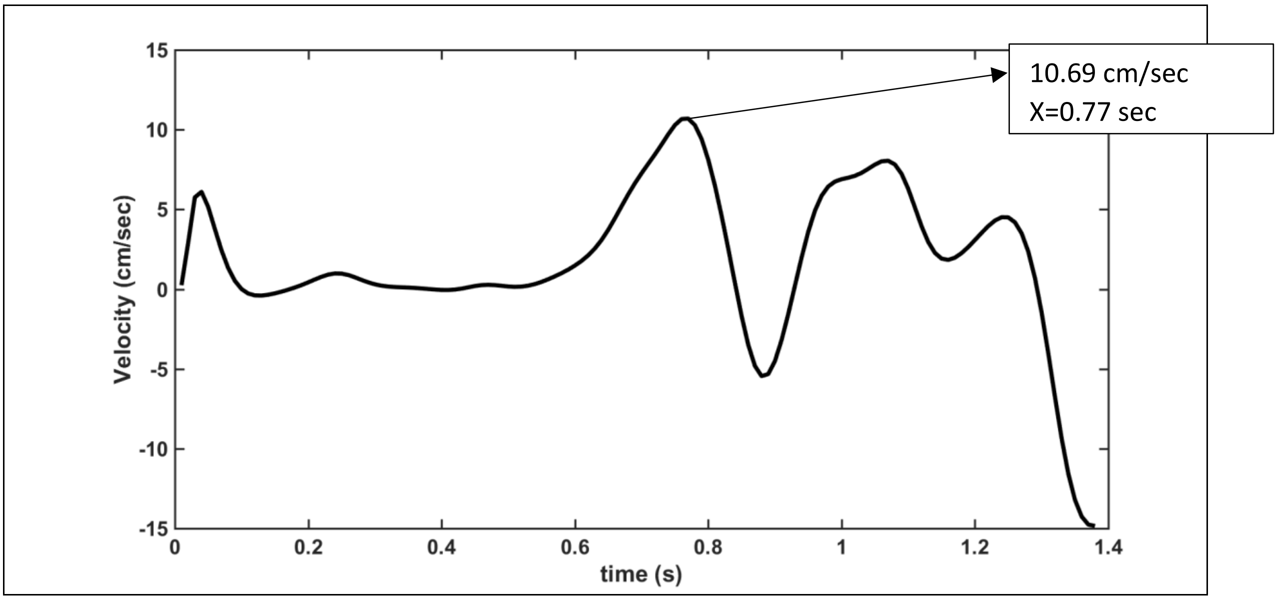

From the peak velocity we travelled forward in time from the max value (10.69 cm/sec) to find first time when the velocity became less than 5% of its max value, that's 0.5345 but here the value it is obtained is at 8th value from the max velocity

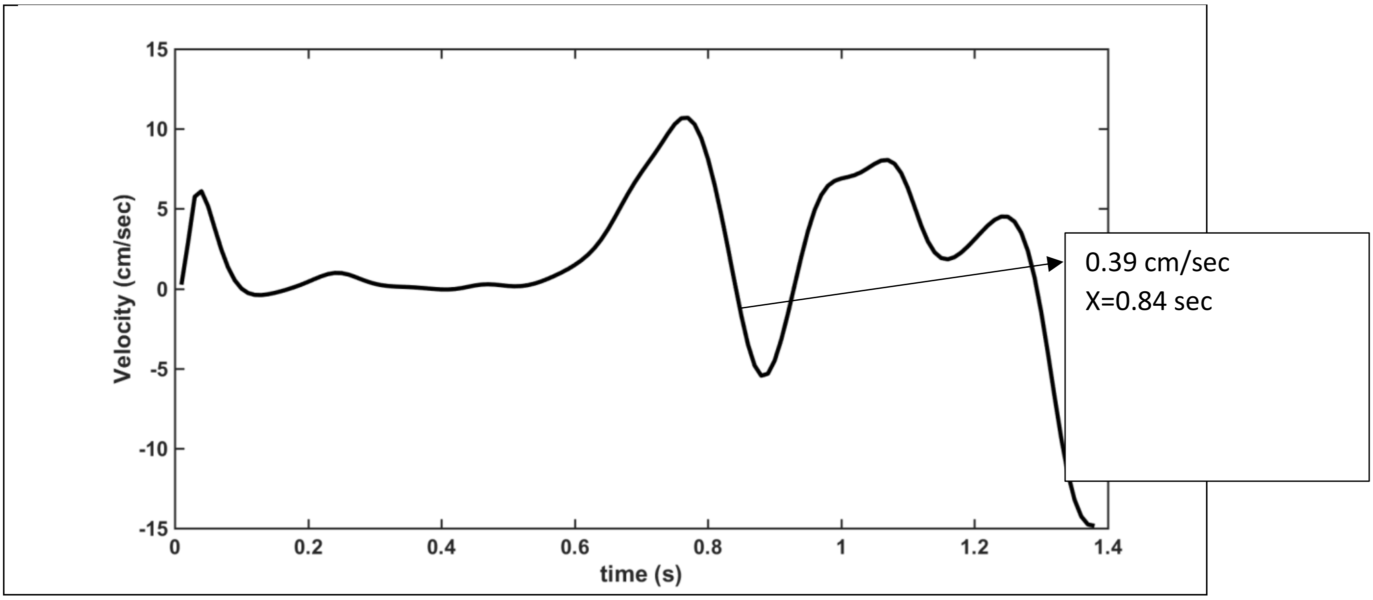

$t_{ed} = t_{max} + 8 = .77 + .08 = .85 - 0.01 = 0.84$ sec

3.64 ~ disp(0.84) = 3.64 ~ 2.27 = 1.37>0.1

Then from 0.84 sec once again algorithm is repeated until the comparison value becomes less than 0.1.

At 54th value it became less than 0.05 max of the velocity profile

$t_{ed} = t_{max} + 0.54 = .77 + .54 = 1.31 – 0.01 = 1.3$ sec

3.56 ~ disp(1.3) = 3.56 ~ 3.561= 0.061<0.1. Therefore 1.3 sec is the end of movement.

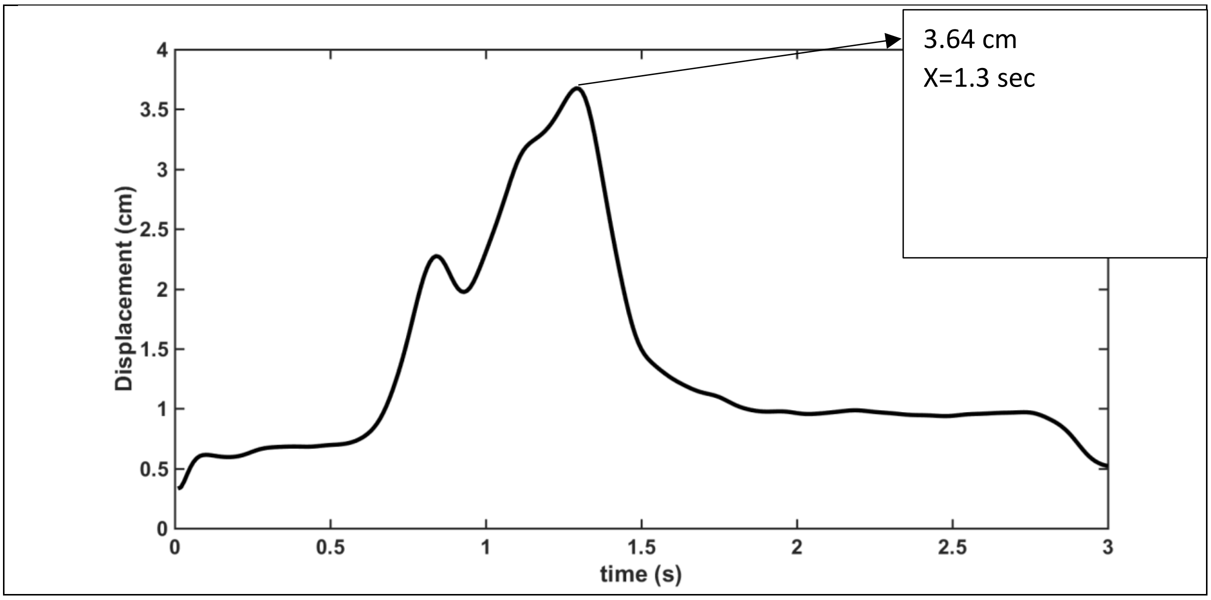

Supplement: Supplemental Information 2 — Movement segmentation algorithm described in detail with examples of how the algorithm segregates movements. [file peerj-06-5763-s002.pdf]
